# The Influence of Porphyromonas Gingivalis Bacterium Causing Periodontal Disease on the Pathogenesis of Rheumatoid Arthritis: Systematic Review of Literature

**DOI:** 10.7759/cureus.4775

**Published:** 2019-05-28

**Authors:** Albertas Kriauciunas, Alvydas Gleiznys, Darius Gleiznys, Gintaras Janužis

**Affiliations:** 1 Dental and Maxillofacial Orthopedics, Lithuanian University of Health Sciences, Kaunas, LTU; 2 Maxillofacial Surgery, Lithuanian University of Health Sciences, Kaunas, LTU

**Keywords:** rheumatoid arthritis, porphyromonas gingivalis, bacterium, periodontal disease, periodontitis, prosthodontics

## Abstract

Periodontal pathologies are highly widespread throughout the world. Epidemiological studies have shown that as much as 1% of the population is suffering from periodontal disease. In recent years, there has been a growing number of studies linking these diseases with autoimmune diseases, especially with rheumatoid arthritis. This literature review evaluates changes in the relationship between periodontal pathologies caused by the bacterium Porphyromonas gingivalis and rheumatoid arthritis. The systematic review of the literature was performed according to the PRISMA analysis protocol. The review was performed with articles from the PubMed database. Searched articles were not older than 5 years. Only full texts and research performed with people were selected. A total of 56 results were received. A review and analysis of their full texts have been carried out and 10 articles were selected according to the established criteria. They were analyzed and results were presented. The results obtained from the literature were based on the influence of Porphyromonas gingivalis on the pathogenesis of rheumatoid arthritis. In the literature, the activity of this bacterium is explained by the analysis of its enzyme peptidylarginine deiminase and its principle of action. Studies have also been found to prove the presence of Porphyromonas gingivalis not only in the oral cavity but its DNA is also found in synovial fluid and plasma. In the researched articles, direct links between Porphyromonas gingivalis and rheumatoid arthritis have led doctors to draw attention to patients' oral hygiene and the condition of parodentium, as this may be the cause of autoimmune lesions. Treatment of periodontal disease will not only help maintain a healthy oral cavity but prevent the spread of bacteria to the surrounding tissues.

## Introduction and background

Rheumatoid arthritis (RA) is an autoimmune disease, which is very common and it affects about 1% of the total population [1]. In the United States, epidemiological studies conducted in 2010 show that RA affects up to 1.3% of the country's population [2]. The prevalence of RA can be due to its wide etiology: genetic factors - HLA-DRB1 allele, obesity, hormonal influences (the majority of patients with rheumatoid arthritis are females), environmental pollutants (pesticides), harmful habits, and cases of rheumatoid arthritis caused by periodontological pathologies in our review. Periodontal diseases (PD) in society are also particularly prevalent. When PD is found, bacteria that can damage the surrounding tissue, alveolar bone, and periodontal ligament persists in the mouths of patients. During these events, the patient loses his teeth. The PD-causing bacteria are Porphyromona gingivalis, Prevotela intermedia, Tannerella forsythia, and Aggregatibacter actinomycetemcomitans. These bacteria are also found in serum and synovial fluid in the joints of patients suffering from RA [3-4]. Amongst all of these pathogens, scientific studies have shown that Porphyromonas gingivalis indirectly causes inflammatory reactions in the body like RA. This persistent pathogen produces an enzyme called peptidylarginine-deiminase (PAD). This enzyme transforms the amino acid L-arginine into citrulline. The transformation occurs when the enzyme destroys the amino group in l-arginine and citrullination of the RA autoantigen takes place (e.g., fibrinogen). It provokes an immune response and antibodies that act against citrullinated protein antigens are produced. These antibodies are called anti-citrullinated protein antibodies (ACPA). These react with antigens containing citrulline (which is a non-standard amino acid produced after post-translational modification of arginine). Since 2010, the American College of Rheumatology has included ACPA in the list of serological markers for RA. Hence, in case of suspicion of the RA disease, a citrulline antibody test is performed, which results in confirmation or rejection of the RA diagnosis [5]. The suggested pathway of pathogenesis is the production of citrullination enzymes, which modifies self-antigens to unrecognized proteins that induce an immune reaction [6]. For the past 15 years, auto-antibodies present in RA patients are citrullinated fibrinogen, vimethine, and alpha-enolase [7].

## Review

Tasks

1. To review and analyze literature on the relationship between rheumatoid arthritis and periodontal disease.

2. To review and analyze the literature on the influence of the enzyme PPAD produced by the bacteria Porphyromonas gingivalis causing inflammation in the joints.

3. To review and analyze the literature on the human-induced changes that link the Porphyromonas gingivalis bacteria to the pathogenesis of rheumatoid arthritis.

Criteria for selecting articles and search methods and strategy

Analysis of literature was performed using the PRISMA analysis protocol. Articles were selected using the keyword "Porphyromonas gingivalis rheumatoid arthritis." Articles that were not older than 5 years, written in the English language, and published in journals were selected. Only the articles which were published on the PubMed were selected.

During research in the PubMed database, the keyword "Porphyromonas gingivalis rheumatoid arthritis" yielded a total of 128 results. We only used the latest articles in our research, therefore, a filter for 5-year-old articles was applied. A total of 95 articles were left. We used full texts for the analysis, therefore, we also added another filter of full texts. Now 92 articles were left. We were interested in articles describing the research with the human population; therefore, we applied another filter that identifies the literature that describes the work performed with people. Finally, 56 articles were left.

From these articles, we reviewed and selected the literature that researched or discussed the links between Porphyromonas gingivalis and RA (Schedule 1). There were 10 such articles.

Literature was selected and analyzed according to the following criteria: 

1. The research was performed using the human population as subjects.

2. The patients were adults.

3. Any relationship investigated between the RA and PD via the enzymes released by the Porphyromonas gingivalis bacterium PPAD (peptidylarginine deiminase).

4. The data from the articles were selected based on whether or not they demonstrated or denied the dependence of the more frequent occurrence of the RA as an autoimmune disease depending on the PPAD activity of the enzymes released by Porphyromonas gingivalis.

Exclusion criteria: we did not include 46 out of the 56 articles because:

1. The subjects were children, and our analysis was performed with studies in adult patients.

2. The studies were focused on the weakening of the immune response strength in patients with RA. However, the challenges we were analyzing were not discussed.

3. The article described osteoclastogenesis with respect to interleukins (especially interleukin 1).

4. The articles did not discuss PPAD operation.

5. Only the genetic relationships between PD and RA were investigated.

6. Microbiological preventive reviews were performed.

7. Articles were not in English.

Analysis

Relationship between rheumatoid arthritis and periodontal diseases: In the literature review, we found 10 articles to support the persistence of Porphyromonas gingivalis and rheumatoid arthritis as pathologies directly related to each other. This is substantiated by the study of scientists. Laugisch et al. found that the PPAD enzyme is more active in RA patients, and in the RA patients who have a diagnosis of the PD [8]. Hashimoto et al. in their two-year follow-up study of patients showed that patients with PD had greater arthritis activity and were more likely to use methotrexane treatment in the future than those who did not have the PD [9]. Mikuls et al. measured antibodies to Porphyromonas gingivalis in blood and found that RA patients and patients with ACPA positive blood tests were more likely to have a higher incidence of PD diseases [10]. Also, Arvikar et al. investigated serums for IgG antibody activity against Porphyromonas gingivalis and the results showed that RA patients had a much higher Porphyromonas gingivalis antibody response than healthy blood donors [11]. The work by Totaro et al. where five-fold higher levels of Porphyromonas gingivalis DNA were found in the synovial tissue of patients with RA than in the control group, should also be mentioned [12]. Reichert et al. also proved in his work that Porphyromonas gingivalis DNA was found 5 times more often in synovial fluid in RA patients than in healthy subjects [13]. De Smit et al. found that RA patients with severe periodontitis had higher IgG and IgM anti-Porphyromonas gingivalis titres, although this had no effect on the Porphyromonas gingivalis in the dental plaque [14]. Also, the same researchers found that more than twice cases of severe periodontitis were observed in RA patients compared to the group of healthy patients.

Influence of the enzyme PPAD produced by the bacteria Porphyromonas gingivalis, causing inflammation in the joints: The influence of Porphyromonas gingivalis on causing the rheumatoid arthritis is based on the activity of the enzyme PPAD. It is an enzyme secreted by the bacteria responsible for citrullination. This enzyme carries out the transformation of the amino acid arginine to the amino acid citruline. The transformation occurs on the enzyme PPAD protein by replacing the amino acid ketamine group with the ketone group. The body responds to this change as an autoimmune response. According to the Fisher Benjamin et al. and Li et al., antibodies to citrullinated proteins in the human body occur much earlier than the diagnosis of RA is determined [7, 15]. Therefore, it is particularly important to examine the relationship between the PD and RA through the PPAD and citrullination mechanisms. Laugisch et al. have shown that citrullination was more frequent in patients with RA than in the control group [8]. Similarly, the same scientists in their ELISE study, measuring antibody levels against citrullinated epitopes, found that PAD and PPAD activities in RA patients were higher than in the control group. Also, Mikuls et al. measured the antibodies in blood and Porphyromonas gingivalis and found that RA patients and patients with positive ACPA in the blood (anti-citrullinated protein antibodies) had a higher incidence in the PD diseases [10]. Lappin et al. in the ELISA study showed that 6 months after the development of the PD, significant changes in the anti-cyclic citrullinated and anti-Porphyromonas gingivalis titres were observed [16]. These studies, discussed by us, delineate the action of the Porphyromonas gingivalis bacterium through the mechanisms of citrullination and the PPAD enzyme.

Human-induced changes that link the Porphyromonas gingivalis bacteria to the pathogenesis of rheumatoid arthritis: We observed many changes in patients with RA and with Porphyromonas gingivalis with expressed pathology of PD. In the study of Laugisch et al., we observed that the PAD and PPAD activities are higher subsequently in patients with RA [8]. Also, the study of Arvikar et al. shows that analysis of IgG activity against Porphyromonas gingivalis in patients with RA showed a much higher antibody response to this bacteria than in healthy blood donors [11]. Moreover, Okada et al. performed a study, analyzing the IgG levels on Porphyromonas gingivalis by ELISA showed that patients with RA had a much higher anti-Porphyromonas gingivalis and anti-cyclical citrullinated peptide antibody levels than the control group [17]. Totaro et al. in examining the DNA of RA and PD patients found that the RA patients had more than five-fold higher levels of Porphyromonas gingivalis DNA in the synovial tissue than the control group [12]. Reichert et al. analyzing the DNA from the synovial fluid found that patients with RA were carrying Porphyromonas gingivalis DNA more often in both the synovial fluid and the dental v:11.9% and 0.9%, respectively [13]. From these studies, we can conclude that the persistence of Porphyromonas gingivalis can cause changes both in the oral cavity and in the blood or even in the synovial fluid. Therefore, patients with PD disease should also be investigated for general abnormalities as well. Okada et al. showed that patients with RA had much higher levels of anti-Porphyromonas gingivalis and anti-cyclic citrullinated peptide antibodies than the control group [17]. All of these studies support the pathological changes caused by Porphyromonas gingivalis and the presence of rheumatoid arthritis as related pathologies.

The action of the mouth-persistent bacterium throughout the body: Porphyromonas gingivalis is a gram-negative, rod-shaped bacterium that is found in the human oral cavity during periodontal disease. This is a particularly common inflammation of the periodontal tissue, which often does not cause any concern or symptoms to the patient to contact the doctor. During their study, Demmer et al. got results during the independent survey of the First National Health and Nutrition Examination of 2011, which showed that patients who had lost more than 5 teeth or had PD, had a higher incidence of RA [18]. Also, according to data from 2005, in the United States, Australia, and Canada, PD ranged from 15% to 30% in the elderly population [8]. However, the incidence of PD does not depend directly on age. PD is the consequence of the accumulation of diseases and illnesses of the whole organism. With the weakening body's immunity and resistance, it becomes more susceptible to bacteria and infections, such as Porphyromonas gingivalis, which are found in people‘s mouths more actively. This bacterium produces an enzyme called peptidylarginine deiminase (PAD, which is specifically identified in the literature of this bacterium, and may be called PPAD). This enzyme carries out the transformation of the amino acid arginine to the amino acid citruline. The transformation occurs on the enzyme PPAD protein by replacing the amino acid ketamine group with the ketone group. The body responds to this change as an autoimmune response. According to Fisher Benjamin et al., citrullinated protein antibodies are manifested much earlier in humans than the diagnosis of RA is made [7]. Therefore, it is particularly important to examine the relationship between the PD and RA through the PPAD and citrullination mechanisms.

In 2015, Laugisch et al. conducted a study to evaluate the activity of the PAD enzyme of the bacteria Porphyromonas gingivalis in the gingival crevicular fluid of humans - an inflammatory exudate that can be collected at the gingival margin or within the gingival crevice, which can then be examined for pathogens present there [8]. This study was conducted on patients with RA and PD, by measuring antibody levels against anti-citrullinated epitopes (also known as antigenic determinants, which are components of antigens identified as antibodies). The measurement was performed by ELISA. The study found that PPAD and PAD activity is higher in patients with RA or periodontal disease than in the control groups (p = 0.038 and p = 0.004, respectively). This indicates the activation of this enzyme due to the bacterial activity, which in the epithelial cells can also carry out citrullination in further tissues. 

Also, Hashimoto et al. in their study examined 72 patients who had never been treated with glucocorticoids or anti-rheumatic drugs [9]. These patients complained of arthralgia (a joint pain due to inflammation) and determined the PD status. A smear was taken from each patient's mouth and the presence of Porphyromonas gingivalis was checked. Patients were followed for 2 years. The comparison was made whether the methotrexate treatment had any effect on patients with or without PD, or Porphyromonas gingivalis [9]. The results showed that patients with PD had higher arthritis activity and a higher probability of subsequent treatment with methotrexate than patients without PD (p = 0.03). However, this study has shown that RA tends to occur in patients with PD but not necessarily Porphyromonas gingivalis. Individuals with pre-RA have significant inflammatory periodontal involvement [9]. Bello-Gualtero et al. got results from their cross-sectional study that there was a significant association between IgG against Porphyromonas gingivalis and ACPAs in pre-RA and markers of RA activity in individuals with eRA [19].

There are cases that show that the patient's RA symptoms are reduced after periodontal treatment. Salemi et al. in 2014 described a case in which a 61-year-old man with RA had arrived at the clinic [20]. His activity according to EULAR/ACR was 8/10 points (> 6 points are sufficient to diagnose RA). His oral cavity examination revealed that he had a lot of periodontological problems - 46 dental roots were to be repaired and many periodontological pockets in the upper jaw were deeper than 5 mm. After 6 months of treatment, the patient's periodontal pockets were not deeper than 3 mm. No focal periodontal infection was observed. As a result of these therapies, the patient had already said that his pain in the joints had improved 1 month later. After 17 months, the ultrasound scan showed complete recovery. From this case, we can see that after tweeting periodontal foci we can stop the inflammatory activity of RA and reduce the damage already done. Therefore, it is particularly important to control the occurrence and frequency of the PD of the population. Also during Shimada et al. research, the serum levels of anti-CCP IgG and anti-PPAD IgG were significantly higher in the RA group than in the non-RA group (p < 0.001 and p = 0.03) [21]. A significant, positive correlation was observed between the serum levels of anti-PPAD IgG and anti-CCP IgG (p = 0.04), but not between the serum levels of PAD-4 and anti-CCP IgG. Also, during Arvikar et al. research, the results showed, that a subset of early RA patients had positive Pg antibody responses [11].

## Conclusions

The results obtained from 10 different authors have shown that PPADs produced by Porphyromonas gingivalis bacterium have a significant influence on the pathogenesis of rheumatoid arthritis. Literature reviewing the relationship between rheumatoid arthritis and periodontal disease has been reviewed and analyzed. The results show that the persistence of the bacteria Porphyromonas gingivalis in the oral cavity can affect the etiology of rheumatoid arthritis. This is based on a higher frequency of patients complaining of rheumatoid and periodontal disease simultaneously. Similarly, research has shown that patients with rheumatoid arthritis got better, or were completely healed, when periodontal foci were removed. The literature reviewing the effect of the enzyme PPAD produced by the Porphyromonas gingivalis bacterium on inflammation in the joints has been reviewed and analyzed. The results showed that the bacterium working through this enzyme disturbs not only the balance of amino acids, but also the whole body's immune system. It unbalances the Ag/Ab complexes and the body produces antibodies against citrullination by auto-antigens. The elements described in the literature affecting Porphyromonas gingivalis in the oral cavity and accompanying patients towards the diagnosis of rheumatoid arthritis were reviewed and analysed. The results showed that the DNA of bacteria Porphyromonas gingivalis is found not only in the serum, but also in the synovial fluid, which supports its influence on the pathogenesis of rheumatoid arthritis.
